# Too fast or too slow?: Considerations in the era of enhanced recovery and FAST Track for upper GI surgery

**DOI:** 10.1007/s00464-026-12894-3

**Published:** 2026-05-14

**Authors:** Florian Seyfried, Matthias Kelm, Felix Nickel

**Affiliations:** 1 Department of General, Visceral, Transplant, Vascular and Pediatric Surgery, University Hospital of Wuerzburg, Würzburg, Germany; 2 Department of General, Visceral and Thoracic Surgery, University Medical Center Hamburg-Eppendorf, Hamburg, Germany

**Keywords:** ERAS, Fast track, Esophagectomy, Gastrectomy

## Abstract

Current ERAS recommendations in UGI surgery are often based on low levels of evidence. While the concepts of enhanced recovery are very promising and should be supported, recent studies have also shown that there might be a need for slowing down for some aspects and components in the era of wide spreading FAST track concepts. Therefore, more high-quality studies are needed to identify key perioperative factors that have a critical impact on postoperative outcomes in UGI surgery, while also not subjecting patients to unnecessary interventions. Importantly, the proportionality, cost-effectiveness, and efficacy of any measures should be critically reviewed to avoid overtreatment and to enhance recovery.

The introduction of the enhanced recovery concept (ERAS, FAST Track) by Hendrick Kehlet has revolutionized perioperative medicine [1]. The combination of various components such as prehabilitation, minimally invasive surgery, short fasting periods, avoidance of prophylactic drains, immediate postoperative extubation, and rapid mobilization has led to increased patient safety, a reduction in perioperative complications, improved patient comfort, and shorter hospital stays [2, 3]. As a result, the FAST Track concept was also rolled out to complex oncological operations on the upper gastrointestinal tract (UGI) and key components of FAST Track surgery have shown to improve perioperative outcomes [4, 5]. However, it has not been demonstrated yet that FAST track relevantly reduces the rate of local complications such as anastomotic leaks, thus, perioperative morbidity remains considerable in oncological UGI surgery [6]. Recent data have shown that even in specialized centers anastomotic leaks still frequently occur and are reported to exceed 10 or even 20% [7, 8]. Despite considerable improvements in the management of anastomotic leaks being established over the recent years, its occurrence can still be devastating when failure-to-rescue occurs [9].

Early detection of local and systemic complications is of great importance and requires a consistent, algorithmically adapted approach. Importantly, appropriate deviations from perioperative standards in order to initiate early treatment and avoid decompensation of the patient are critical since local and systemic complications can quickly lead to a chain of subsequent perioperative morbidity and failure-to-rescue [10]. Therefore, it is desirable to identify effective perioperative measures which prevent anastomotic leakage. Historically, long periods of fasting and consistent drainage of the conduit via nasogastric tubes had been recommended to reduce tension and improve blood flow at the anastomotic region by decreasing gastric wall distension after esophageal resections. Modern FAST track concepts, which are at least partially based on low or moderate evidence, abandon these measures. In contrast, due to the persistently high rate of local complications, perioperative measures contradicting the idea of FAST track concepts have also been evaluated to improve short-term outcome. A recent prospective randomized trial has shown that consequent postoperative nasogastric tube decompression of the gastric conduit for five days after esophageal resection surgery led to a lower rate of anastomotic leakage than early removal of the nasogastric tube [11]. Furthermore, it has also been demonstrated that establishing prophylactic drainage after gastrectomy can reduce postoperative reinterventions and re-operations [12]. Other strategies such as preemptive endoluminal vacuum therapy to reduce anastomotic leakage rates after esophageal surgery have also been evaluated but currently lack clear evidence to support this concept [13]. In line with those ideas, Zang et al. followed the approach of preclinical studies indicating that hyperbaric oxygen therapy could improve anastomotic healing [14]. While the exact pathophysiologic mechanism remains unclear, it appears that an enhanced oxygen supply of the anastomotic area improves the healing process. Previous studies on colorectal anastomosis confirmed this observation and identified local hypoxia as a relevant risk factor for anastomotic leakage due to changes of metabolic homeostasis. In line with that, Zang et al. demonstrated in their recent retrospective study after Propensity Score Matching that prolonged postoperative mechanical ventilation (> 24 h) was associated with improved oxygen index and a significant decrease in anastomotic leak rates following McKeown esophagectomy. While the idea is interesting and data well presented, the manuscript has important limitations and raises critical questions. On the one hand, the experience of the operating surgeon, which has a relevant effect on the perioperative outcome, is not presented. In addition, the concept of prolonged ventilation might not be considered in general but maybe for a small cohort of patients with relevant pulmonary comorbidities or insufficient breathing activity. Thus far, other retrospective clinical studies investigating whether postoperative oxygen therapy reduces the incidence of anastomotic leaks remain controversial [15]. However, a single prospective randomized trial demonstrated that increased perioperative oxygen supply for 6 h after gastric cancer surgery reduced anastomotic complications. While the concept of prolonged postoperative mechanical ventilation is a complete contradiction to all established Fast Track and ERAS concepts, we thus seem to be at a stage where some of the elements of such concepts should be revisited and further refined for optimal organ-specific strategies for UGI surgery.

Sufficient gastric conduit perfusion and consecutive oxygenation are pivotal for anastomotic healing. In this context, the use of extended intraoperative perfusion measurement and imaging technologies (e.g., hyperspectral imaging) and principles of ischemic conditioning to improve gastric conduit perfusion have also been introduced [16]. However, these measures have thus far not demonstrated convincing effects on leakage rates. Moreover, the incidence of pulmonary infections and pleural effusion was also reduced in the study of Zang et al. with prolonged postoperative mechanical ventilation. The authors argue that prolonging postoperative mechanical ventilation declines early oral intake and may therefore have reduced occult aspiration. Following their argumentation, they conclude that occult aspiration might contribute to an increased incidence of anastomotic leakage. However, while the explanation seems conclusive, the authors’ perioperative management allowed the introduction of fluid and solid food only on the 11th day after surgery, following a computer tomography scan and iodine contrast examination without signs for anastomotic leakage. While this strategy is a radical contrast to established enhanced recovery concepts, it raises at least further questions about the meaningfulness and effectiveness of single FAST Track concept elements in the vulnerable cohort of patients receiving UGI surgery. While the currently debated results can be seen es exploratory rather than confirmatory this should lead to further investigations that hopefully lead to more organ-specific and tailored recommendations.

Overall, current ERAS recommendations in UGI surgery are often based on low levels of evidence. While ERAS concepts are very promising and should generally be supported, recent studies have also shown that there might be a need for slowing down for some aspects and components specifically in UGI surgery. Therefore, more high-quality studies are needed to identify key perioperative factors that have a critical impact on postoperative outcomes in UGI surgery with organ-specific trials and recommendations of the critical ERAS concepts, while also not subjecting patients to unnecessary interventions. Importantly, the proportionality, cost-effectiveness, and efficacy of any measures should be critically reviewed to avoid overtreatment and to truly enhance recovery after surgery.
